# Care of Terminally Ill Cancer Patients: An Intensivist’s Dilemma

**DOI:** 10.4103/0973-1075.68406

**Published:** 2010

**Authors:** Sukhminder Jit Singh Bajwa, Sukhwinder Kaur Bajwa, Jasbir Kaur

**Affiliations:** Department of Anaesthesiology and Intensive Care, Gian Sagar Medical College & Hospital, Ram Nagar, Banur, Punjab, India; 1 Department of Obstetrics & Gynecology, Gian Sagar Medical College & Hospital, Ram Nagar, Banur, Punjab, India

**Keywords:** Cancer, Intensive care, Mechanical ventilation, Respiratory failure, Terminally ill

## Abstract

**Background and Context:**

Treatment of terminally ill cancer patients always poses great challenges especially when these critical patients are admitted in intensive care unit (ICU). The severity of their diseases throws a clinical and ethical dilemma to the treating intensivist.

**Aims and Objectives:**

To evaluate the benefits of intensive care treatment in terminally ill cancer patients and also to find out whether optimal utilization of critical care resources has got any positive financial, psychological and clinical outcome.

**Materials and Methods:**

A retrospective evaluation of 53 terminally ill cancer patients, who got admitted to ICU of our department, was carried out. Majority of these patients presented with terminal phase of illness involving multi-organ pathologies with diverse range of symptoms. These patients were provided ventilatory, symptomatic and supportive treatment on patient-to-patient basis. Strict and vigilant monitoring of all vital parameters was carried out. At the end of study, all the data was compiled systematically and was subjected to statistical analysis using non parametric tests.

**Results:**

The demographic profile of such patients was highly variable with regard to educational, social and financial status (*P*<0.05). The most common group of cancer was hematological malignancies (24.53%) followed by lung cancer (18.87%), uteri-ovarian (15.09), colorectal (13.2%) and others. Significant number (*P*<0.05) of patients (64.15%) required mechanical ventilation and ionotropic support (79.24%). Mortality increased with increasing number of organ system involvement and reaching up to 100% with involvement of 5 or more organ systems.

**Conclusions:**

ICU care is the best form of treatment for terminally ill but resources should be used optimally so that a young deserving patient should not be sacrificed for the scarcity of resources.

## INTRODUCTION

The advancements in medical healthcare have achieved greater heights in the last few decades. However, despite the availability of early diagnostic and therapeutic interventions, the number of deaths in cancer patients is rising day by day. This scenario is more commonly observed in the developing nations like India where majority of deaths due to carcinogenic diseases go unrecognized.[1–3]

The word cancer was a taboo in the past, especially in the last four or five decades, which always invariably meant an incurable disaster and certain death of the patient. Today, with advancements in the medical fields especially with the growth of the oncological sciences, hopes and faith have been instilled again into the mind of the masses about the curative and palliative aspects of various cancers. Though the number of cancer patients may have increased over the last decade but that can be mainly attributable to the early and accurate diagnosis of various carcinogenic diseases.[4] As such, the load of cancer patients in the various higher centers is increasing day by day and majority of such patients have to be admitted in general critical care units rather than in the specialized oncology centers. The intensivists, anesthesiologists and the physicians have to manage such cases and in doing so they have to live up to new challenge to treat such cases. They have to keep themselves updated all the times regarding various treatment regimens and intervention strategies while treating such patients as oncologist’s services are not uniformly available in majority of these centers.

The tertiary care centers and especially the oncologic centers have to attend a large number of cancer patients, at varying stages of cancer, either on OPD basis or as an emergency patients. These categories of patients are entirely different from the normal patients as majority of them do know about the diagnostic and prognostic implications of their disease. Handling of such patients is not just a clinical difficulty but also a socio-cultural and behavioral challenge. The clinician has to be precise not only in his clinical skills but also should have some special attitudinal and behavioral constructs while treating such patients.

These situations become all the more challenging when the physician has to deal with the terminally sick patients who are almost at the far end of their disease process and are knocking on the doors of eternity. These patients are either diagnosed late or have been on anti-cancer regimen for a prolonged duration. Majority of them hardly visits a physician during the clinically asymptomatic phase of their respective oncologic disease. It is only during the clinically expressed disease entity or some bodily discomfort that force them to see a physician. By that time, the oncogenic process would have got distributed widely and would have invaded a lot of other tissue structures of the body. This leads to an irreversible stage from where it becomes almost impossible for a diseased body to stage a comeback to its normal.

At this stage, patients come to the hospital with so many expectations from the clinician and do expect a little magic to ward off their irreversible disease stage. The expressions in their blank eyes demand an altogether urgent attention from the attending doctor with a hope that they will get another lease of life for some more years. This acquires all the more a gigantic task for physician when these patients report with multiorgan involvement especially the invasion of pulmonary, cardiac, hepatic and renal tissues. The prognosis is almost nil with multiorgan disease and one can only provide palliative care.[5] The biggest clinical challenge to an intensivist at this stage is whether his services will be of any benefit to the patient. While putting such patients on mechanical ventilation, which has got massive pulmonary involvement, is always the biggest dilemma for an intensivist knowing very well that the intervention is not going to improve the patient’s underlying disease condition. Rather prolonged ICU stay will add to the economic burden of the financially depleted relatives who have been spending a lot for the treatment of underlying cancer for quite a while now. But ethically and socially neither the relatives nor the intensivist will like to aggravate the misery and agony of such patients who cannot survive without artificial ventilation for even a day. To avoid the feeling of guilt due to social and moral pressures, even though knowing the fate of the patients in majority of the cases, relatives and attendants almost always request for the ventilatory support despite having a financial crunch. Though the mortality index of ICU increases with such admissions but an intensivist has to support such care and intervention on the basis of socio-cultural and ethical considerations. The other big dilemma for an intensivist is that he cannot provide good results among such patients and to some extent it does cast a negative psychological impact on the relatives and attendants of the patient. By admitting such patients in ICU, it becomes very challenging task for an intensivist to live up to the faith of the people. An intensivist has to be very straight forward in his or her approach at this stage and at the same time he or she has to handle the personals in a very delicate and tactful manner; otherwise the image of ICU will just be limited to the projection of a mortality ground to the general public. This aspect acquires a great importance as most of the population in the developing countries like ours, is not properly educated as well as not well informed about the delicate intricacies of intensive and tertiary care.

With increasing awareness, medical advancements, realization of value of human life and increasing availability of intensive care facilities, the role of intensivist has become more and more tough and challenging and burden on his mind and shoulders increases in direct proportion to the public expectations. The role of an intensivist acquires a dual model and that not just of a sound clinician but of a good diplomat as well.

Keeping the various implications of tertiary care of terminally ill cancer patients, we undertook a retrospective study of fifty-three patients of our institute who were admitted to ICU with terminal stages of various cancers with an aim to evaluate the benefits of intensive care treatment in terminally ill cancer patients and also to find out whether optimal utilization of critical care resources has got any positive financial, psychological and clinical outcome.

## MATERIALS AND METHODS

After the clearance from institutional ethical committee, we undertook a retrospective evaluation of 53 terminally ill cancer patients, who got admitted to ICU of our department from the period of 20^th^ December 2006 to 20^th^ April 2010. 46 out of the 53 patients were established cases of malignancy who were already taking anticancer regimens for quite a long time from different oncology centers while rest were diagnosed for the first time in the institute only. Majority of these patients presented with features of shock, restlessness, altered sensorium, gastro-intestinal bleed, pain abdomen, severe backache and arrhythmias. Few of these patients presented with symptoms of advanced respiratory failure and were in urgent need for mechanical ventilation. Ventilatory support was given to 34 of these patients while rests of the patients were treated without ventilatory support. Besides the ventilatory support, the symptomatic and supportive treatment was carried out in close coordination with the oncologists and the physician on patient to patient basis. During the ICU stay, all the relevant investigations were carried out for ascertaining the stages of the malignancy and the extent of organ involvement. The ICU treatment comprised of specialized anticancer regimens, mechanical ventilation, monitoring of vital parameters, blood, platelets and plasma transfusion, electrolyte and metabolic correction, parenteral and enteral nutrition, hemodialysis, management of co-morbid diseases, psychological counseling of patients and their relatives besides routine antibiotic prophylaxis and other supportive medications.

The consent of the relatives was taken regarding the poor prognosis and the expenses involved while treating such patients. Intensive monitoring was carried out for all the patients during their stay in ICU which included monitoring of heart rate (HR), electrocardiogram (ECG), non-invasive and invasive blood pressure, end tidal carbon dioxide, pulse oximetry, central venous pressure monitoring, temperature etc. Particular attention was paid to the acute side effects of anticancer regimen being administered to the patients. At the end of the study, all the statistical data including patient demographics, indications for ICU admission, type of malignancy and stages of malignancy, organ involvement and patient outcome were organized systematically and subjected to statistical analysis with non parametric tests like chi square test. Value of *P*<0.05 was considered as significant.

## RESULTS

From 20^th^ December 2006 to 20^th^ April 2010, there were 879 ICU admissions, including 53 admissions pertaining to the terminally ill cancer patients. Most of these 53 cancer patients came to the hospital for the first time and they were receiving anticancer treatment from other centers. Most of these patients came to the emergency ward due to the nature of their presenting symptoms and were subsequently shifted to ICU for further management.

The demographic profile of the patients is shown in Table 1.

**Table 1:** Demographic profile of the patients

Number of patients	53
Age of patients (Mean ± SD)	58.18±10.38
Educational level	Illiterate-4 (7.54)
	Up to High School-8 (15.09)
	> High school-41* (77.36)
Financial status (Family income in rupees/month)	<3000-5 (9.43)
	3000-5000-8 (15.1)
	>5000-40* (75.47)
Location	Rural-24 (45.28)
	Urban-29 (54.72)
Diagnosis of the cancer	Known-46* (86.79)
	First time-7 (13.21)
Duration of anticancer treatment(in years) (mean±SD)	2.8±0.64

* (*P*<0.05). Figures in parentheses are in percentage

The average mean age of these patients was 58.18±10.38 and a median value of 61 with a range of 42-88 years of age. The statistical analysis of educational status revealed statistically significant values (*P*<0.05) as 77.36% of the patients had educational status of more than a high school, 15.09% had education up to 10^th^ standard while 7.54% of the patients had almost negligible educational level rather they were illiterate. The 9.43% of patients were having income below Rs 3000/month which is almost equivalent to below poverty line while those having income between Rs 3000-5000 constituted 15.1% of the total patients. 75.47% of the patients had family income greater than Rs 5000/month which was highly significant statistically (*P*<0.001). 45.28% of the patients who got admitted in critically sick condition hailed from rural area while 54.72% of the population comprised of urban society which on statistical comparison turned out to be a non-significant relation (*P*>0.05). The percentage of population (86.8%) was statistically significant (*P*<0.05) who were established cases of various cancer and had been on anti-treatment for a long time and the mean duration of the treatment was 2.8 years in such patients.

The most common reasons for admission to ICU are shown in Table 2.

**Table 2:** Reasons for admission to intensive care unit

Primary indication	Number of patients
Respiratory distress/failure	21 (39.62)
Gastro-intestinal symptoms	17 (32.07)
Heart Disease/CVS instability	7 (13.20)
Hematemesis and hemoptysis	5 (9.43)
Neurologic disorders	3 (5.66)

The most common primary indication for admission to ICU was respiratory insufficiency (39.62%) followed by gastro-intestinal symptoms like severe anorexia, nausea, vomiting and pain abdomen (20.75%). Heart disease and cardiovascular instability was responsible for 13.20% of the total cancer admissions in ICU while Haematemesis/hemoptysis and neurologic disorders accounted for 9.43%/5.66% admissions respectively.

Table 3 shows the underlying cancer disease which necessitated the admission of these patients to ICU. Hematological malignancies were the most common cancer group in these patients (24.53%) while pulmonary, ovarian, colorectal, gastro-esophageal, prostatic etc were the other cancer pathologies which the patients were suffering from and are tabulated in descending order.

**Table 3:** Underlying cancer disease

Types of cancer	Number of patients (%)
Hematological malignancies	13 (24.53)
Lung/bronchiogenic carcinoma	10 (18.87)
Ovarian/uterine/cervical	8 (15.09)
Colorectal	7 (13.2)
Esophageal/gastric	5 (9.43)
Prostatic/testicular	3 (5.66)
Pancreatic	2 (3.77)
Breast	2 (3.77)
Renal/urinary bladder	2 (3.77)
Thyroid	1 (1.89)

Airway protection became necessary in 64.15% of the patients as shown in Table 4, who were intubated and subsequently put on mechanical ventilation which on statistical analysis turned out to be a significant value (*P*<0.05). Requirement of ionotropic support became essential for the maintenance of hemodynamic stability in significant percentage (79.24%) of cancer patients. The mean total duration of stay in ICU was 38.34 days with standard deviation of 5.66 and the range varied from minimum 5 days to maximum 71 days. Mortality was significantly higher among these patients (77.36%) as we could not save 42 patients among 53 most of whom had developed multiorgan failure.

**Table 4:** Patients data

Treatment pattern in ICU	Number of patients
Mechanical ventilation	Required-34* (64.15)
	Not required-19 (35.85)
Ionotropic support	Required-42* (79.24)
	Not required-11 (20.76)
Stay (days) in ICU (mean ± SD)	38.34±5.66
Outcome (death/survival)	41*/12 (77.36/22.64)

* (*P*<0.05)

The mortality figures climbed northward as number of organ involvement increased in the disease process. As shown in Table 5, three patients out of six succumbed to their disease that had just a single organ involvement while binary organ dysfunction led to the deaths of 61.5% of the patients. Mortality increased significantly (*P*<0.05) with progressive involvement of multiple organs as shown in the Table number 5. Mortality rate increased to 80% with three, 91.67% with four and 100% deaths with five or more organs getting involved in the disease process.

**Table 5:** Patients data

Number of organs involved at the time of admission	Number of patients	Number of deaths and % of mortality
One	6	3 (50)
Two	13	8 (61.5)
Three	15	12 (80)*
Four	12	11 (91.67)*
Five or more	7	7 (100)*

* *P*<0.05

## DISCUSSION

Throughout the world, cancer patients do require ICU admission at the terminal stages of the disease or sometimes even much earlier. Majority of our patients were well educated and did know about their disease and its stage to a large extent which also helped us in pre-admission counseling and in explanation of the prognosis to them and to their relatives. Anti-cancer treatment was already being administered to a significant proportion of the patients for quite a long time from different oncologic centers. The financial status of majority of these patients was good which enabled them to carry on the treatment of cancer for quite a long time and they all came from different strata of rural and urban background which clearly establishes the fact that cancer is prevalent almost equally in all the sections of the society irrespective of its geographical location.

The respiratory failure is one of the most common presenting symptoms in patients with terminal stages of cancer and sometimes the only indication for admission to ICU as was also discovered in our retrospective analysis. Majority of the patients who got admitted with non-respiratory symptoms did eventually develop respiratory failure necessitating artificial ventilation. The severity of respiratory disease at the time of admission to ICU and the urgency of mechanical ventilation is associated with a very high mortality.[6] The usual score patterns, hematological picture and type of cancer are hardly of much significance in terminally ill cancer patients in evaluating the prognosis. Similarly, the gastro-intestinal, cardiac and other symptoms are hardly in practice to evaluate accurately the prognostic stages of such patients. The spectrum of cancer disease is very wide, as is evident from our analysis, and it is not the type of cancer that warrants ICU admission, rather it is the stage of cancer which determines the severity and necessity of intensive care intervention.[7–10] The main determinants in such cases are basically the number of organs involved at the time of admission as well as rapidly progressive involvement of multiple-organ involvement during ICU stay. We experienced a very high mortality incidence with increasing involvement of organ systems as the figures touched the century percentage mark with involvement of five or more organs.[11,12]

Pneumonitis is one of the most common underlying respiratory diseases in cancer patients. Though cancer and pneumonia do not warrant ICU admission but delay in treating disease can cause perfusion-ventilation mismatch.[13] The resulting need for mechanical ventilation is itself a poor prognostic factor[14,15] and 32 out of 34 of our patients who required mechanical ventilation never came off from the ventilator and ultimately expired. Ionotropic support became necessary in 80% of the patients and among them 39 patients succumbed to the clutches of fatal hemodynamic instability. The dilemma for us increased further when relatives of the few patients did not give us consent to use mechanical ventilation rather requested to continue with the pain free and palliative aspect of cancer treatment. The sequences of events leading to respiratory failure are being seen as the terminal stages of the oncogenic process by most of the oncologist. The dilemma begins when most of the ICU admissions for such patients are denied across the world as most of the intensivists feel that invasive ventilation is a strong predictor of mortality in such patients. Occupancy of the beds by such patients leads to denial of ICU admission to so many deserving and salvageable patients. The scarcity of intensive care facilities and the services of well trained intensive care staff do warrant the careful and optimal use of these facilities for the betterment of mankind.

These scarce facilities along with high cost involved in the mortality of such patients have been on the prime agenda of various health analysts who have decried the high cost of dying.[16–21] The equal inability of an intensivist to produce perfect results in such cases not only traumatizes the family members psychologically and physically but it causes a big hole in their pockets leading to the financial depletion. Nowadays suitable measures are being adopted not just in developing countries but by developed nations as well to control the costs involved in futile care at the end of life.[22]

The poor prognosis of ventilated patients with cancers, especially hematological malignancies, has been a major incentive in the use non-invasive ventilation in such patients. While experience in non invasive ventilation is of prime importance, it is the early use especially in conditions requiring supplemental oxygen and a drop in SaO_2_ of >10% that the most benefit is expected. We administered non invasive ventilation in 6 of our patients and could save 5 out of them. The survival index among such patients is quite low and it was 22.64% in our setting which further added to the burden of dilemma on our mindset.

Advance directives and hospice services have been developed keeping the compassion and dignity of patients in consideration. The whole exercise basically aims at reserving the scarce intensive facilities as well as controlling the costs involved.[23,24] These moral justifications and economic considerations reflected in advanced directives are beneficial both to the patients as well as to his family.[25,26] These arguments are based on the ground that in patients with advanced cancer death is imminent and whatsoever interventions are done in these patients, it is not going to cure the disease process or decrease the mortality and morbidity. Though these services are very much available in the developed nations, they have yet to make a ground in developing nations like ours.[25]

The dilemma get exaggerated for the intensivist when he has to face the do not resuscitate orders (DNR) for such patients by the superior authority as it is argued that use of resources on such patients are extremely wasteful steps.[27–29] The unpredictability of death in these patients and the non-reliability of any method to estimate the time of death can prolong the ICU stay of such patients leading to increased consumption of scarce resources. Even the outcome of such interventions cannot be established with certainty which further increases the economic burden on patient and his relatives. Though DNR orders are in place for majority of ICU’s but no such orders have been carried out in our institute mainly to preserve the dignity and give respect to the autonomous decision of the relatives as well as living up to the faith shown in us by them as most of them expects us to do all the aggressive efforts to save the life of the patient till the last breath.[30,31] They expect from us to continue the life sustaining procedures even being fully that such interventions are not going to be of much help in their patients who have attained an almost vegetative state.[32,33]

The intensive care services should be appropriately used for such patients keeping in mind the following points thereby decreasing the intensity of dilemma in handling such patients.

- Respect should be given to the decisions and faith of the cancer patients and their relatives.
- The criteria’s for ICU admission should be clearly defined for these patients.
- Not a single young patient who requires ICU admission should be sacrificed on account of non-availability of bed and denied the privileges of intensive care facilities.
- Alternatives to ICU admission facilities should be made available to such patients thus relieving the economic burden on scarce resources.
- The quality of life post-ICU admission should be estimated and evaluated for further medical and ethical decisions concerning the level of care in the ICU.

One alternative is HOSPICE care which is aimed at caring and not curing methods for the terminally ill cancer patients. Quality of left life is given more stress rather than aiming for prolonging the life days. It covers various aspects such as pain management, symptomatic treatment, psychological counseling, training of the family members in the care methods of terminally ill patients and preparing them for the eternal outcome. But this method is yet to pick up the pace in developing nations like ours where there are multitudes of laggards which are hindrances in the successful execution of such projects.

## CONCLUSION

Thus, we can conclude from all this discussions that ICU is still the best place for such treatment modalities in spite of shortage of specialist services in our country. But equal attention must be paid to provide intensive care facilities to the young patients who have got higher chances of survival by optimal and careful use of these scarce facilities.

## References

[1] Magrath I, Litvak J (1993). Cancer in developing countries: Opportunity and challenge. J Natl Cancer Inst.

[2] Gupta PC, Sankaranarayanan R, Ferlay J (1994). Cancer deaths in India. Is the model-based approach valid? Bull World Health Organ.

[3] Mathers CD, Shibuya K, Boschi-Pinto C, Lopez AD, Murray CJ (2002). Global and regional estimates of cancer mortality and incidence by site: I. Application of regional cancer survival model to estimate cancer mortality distribution by site. BMC Cancer.

[4] Jones R, Latinovic R, Charlton J, Gulliford MC (2007). Alarm symptoms in early diagnosis of cancer in primary care: Cohort study using general practice research database. BMJ.

[5] Knaus WA, Draper EA, Wagner DP, Zimmerman JE (1985). Prognosis in acute organ-system failure. Ann Surg.

[6] Hauser MJ, Tabak J, Baier H (1982). Survival of patients with cancer in a medical critical care unit. Ann Intern Med.

[7] Azoulay E, Alberti C, Bornstain C, Leleu G, Moreau D, Recher C (2001). Improved survival in cancer patients requiring mechanical ventilatory support: Impact of non-invasive mechanical ventilatory support. Crit Care Med.

[8] Benoit DD, Vandewoude KH, Decruyenaere JM, Hoste EA, Colardyn FA (2003). Outcome and early prognostic indicators in patients with a hematological malignancy admitted to the intensive care unit for a life threatening complication. Crit Care Med.

[9] Azoulay E, Schlemmer B (2006). Diagnostic strategy in cancer patients with acute respiratory failure. Intensive Care Med.

[10] Soares M, Fontes F, Dantas J, Gadelha D, Cariello P, Nardes F (2004). Performance of six severity of illness scores in cancer patients requiring admission to the intensive care unit: A prospective observational study. Crit Care.

[11] Blot F, Guiguet M, Nitenberg G, Leclercq B, Gachot B, Escudier B (1997). Prognostic factors for neutropenic patients in an intensive care unit: Respective roles of underlying malignancies and acute organ failure. Eur J Cancer.

[12] Azoulay E, Moreau D, Alberti C, Leleu G, Adrie C, Barboteu M (2000). Predictors of short-term mortality in critically ill patients with solid malignancies. Intensive Care Med.

[13] Hilbert G, Gruson D, Vargas F, Valentino R, Gbikpi-Benissan G, Dupon M (2001). Noninvasive ventilation in immunodepressed patients with pulmonary infiltrates. N Engl J Med.

[14] Soares M, Salluh JI, Spector N, Rocco JR (2005). Characteristics and outcomes of cancer patients requiring mechanical ventilatory support for >24 hrs survival. Crit Care Med.

[15] Stauffer JL, Tobin MJ (1994). Complications of translaryngeal intubation. Principles and practice of mechanical ventilation.

[16] Turnbull AD, Carlon G, Baron R, Sichel W, Young C, Howland W (1979). The inverse relationship between cost and survival in the critically ill cancer patient. Crit Care Med.

[17] Ginzberg E (1980). The high costs of dying. Inquiry.

[18] Schroeder SA, Showstack JA, Schwartz J (1981). Survival of adult high-cost patients: Report of a follow-up study from nine acute-care hospitals. JAMA.

[19] Bayer R, Callahan D, Fletcher J, Hodgson T, Jennings B, Monsees D (1983). The care of the terminally ill: Mortality and economics. N Engl J Med.

[20] Scitovsky AA (1984). “The high cost of dying”: what do the data show?. Milbank Mem Fund Q Health Soc.

[21] Scitovsky AA, Capron AM (1986). Medical care at the end of life: The interaction of economics and ethics. Annu Rev Public Health.

[22] Singer PA, Lowy FH (1992). Rationing, patient preferences and cost of care at the end of life. Arch Intern Med.

[23] Lubitz JD, Riley GF (1993). Trends in medicare payments in the last year of life. N Engl J Med.

[24] Lubitz J, Prihoda R (1984). The use and costs of medicare services in the last 2 years of life. Health Care Financ Rev.

[25] d’Oronzio JC (1993). Good ethics, good health economics. New York Times. June 8.

[26] Lundberg GD (1993). American health care system management objectives: The aura of inevitability becomes incarnate. JAMA.

[27] Schneiderman LJ, Jecker NS, Jonsen AR (1990). Medical futility: Its meaning and ethical implications. Ann Intern Med.

[28] Tomlinson T, Brody H (1990). Futility and the ethics of resuscitation. JAMA.

[29] Lantos JD, Singer PA, Walker RM, Gramelspacher GP, Shapiro GR, Sanchez-Gonzalez MA (1989). The illusion of futility in clinical practice. Am J Med.

[30] Gleeson K, Wise S (1990). The do-not-resuscitate order: Still too little too late. Arch Intern Med.

[31] Smedira NG, Evans BH, Grais LS, Cohen NH, Lo B, Cooke M (1990). Withholding and withdrawal of life support from the critically ill. N Engl J Med.

[32] Emanuel LL, Barry MJ, Stoeckle JD, Ettelson LM, Emanuel EJ (1991). Advance directives for medical care–a case for greater use. N Engl J Med.

[33] Steinbrook R, Lo B, Moulton J, Saika G, Hollander H, Volberding PA (1986). Preferences of homosexual men with AIDS for life-sustaining treatment. N Engl J Med.

